# Genetic Differentiation of a New World Screwworm Fly Population from Uruguay Detected by SNPs, Mitochondrial DNA and Microsatellites in Two Consecutive Years

**DOI:** 10.3390/insects11080539

**Published:** 2020-08-16

**Authors:** Luana Walravens Bergamo, Karina Lucas Silva-Brandão, Renato Vicentini, Pablo Fresia, Ana Maria Lima Azeredo-Espin

**Affiliations:** 1 Departamento de Genética, Evolução, Microbiologia e Imunologia, Instituto de Biologia, Universidade Estadual de Campinas (UNICAMP), Campinas SP 13083-970, Brazil; shinapes@unicamp.br; 2 Programa de Pós-Graduação em Genética e Biologia Molecular, Universidade Estadual de Campinas (UNICAMP), Campinas SP 13083-862, Brazil; 3 Centro de Biologia Molecular e Engenharia Genética, Universidade Estadual de Campinas (CBMEG-UNICAMP), Campinas SP 13083-875, Brazil; klsilva@gmail.com; 4 Centro de Ciências Naturais e Humanas, Universidade Federal do ABC (CCNH-UFABC), Santo André SP 09210-580, Brazil; 5 Unidad Mixta Pasteur + INIA (UMPI), Institut Pasteur de Montevideo, Montevideo 11400, Uruguay

**Keywords:** effective population size (N_e_), population genomics, genotyping-by-sequencing (GBS), insecticide resistance, population structure

## Abstract

**Simple Summary:**

The New World screwworm (NWS) fly is a pest related to economic impacts in livestock breeding, mainly because females lay their eggs in wounds and natural cavities of warm-blooded vertebrates. A direct way to control this pest is the sterile insect technique (SIT), which is based on the release of sexually sterilized males to mate with wild females, resulting in the absence of new flies. The first step to the implementation of such program is to know how flies’ populations are structured, that is, if they can freely mate in the field or, otherwise, if they are impeded of mate due to spatial or temporal barriers. Here we investigated genomic polymorphisms to infer temporal structure in a population of the NWS fly from Uruguay in two consecutive years (approximately 18 generations). We found that this time was enough for this population to change its genetic composition, but it was not sufficient to prevent free mate among individuals from one year to the next. We also found signal that some of the polymorphisms are related to the response of the population to insecticide chemical control. This approach can be used in the future to estimate spatial barriers to geographically isolated populations.

**Abstract:**

The New World screwworm (NWS) fly, *Cochliomyia hominivorax* (Diptera: Calliphoridae), is an economically important ectoparasite currently distributed in South America and in the Caribbean basin. The successful eradication of this species in USA, Mexico and continental Central America was achieved by a control program based on the sterile insect technique (SIT). In order to implement a genetic control strategy over the NWS fly’s current area of occurrence, first, it is necessary to understand the species dynamics and population structure. In order to address this objective, the spatial genetic structure of the NWS fly was previously reported in South America based on different genetic markers; however, to date, no study has investigated temporal changes in the genetic composition of its populations. In the current study, the temporal genetic structure of a NWS fly population from Uruguay was investigated through two consecutive samplings from the same locality over an interval of approximately 18 generations. The genetic structure was accessed with neutral and under selection SNPs obtained with genotyping-by-sequencing. The results gathered with these data were compared to estimates achieved with mitochondrial DNA sequences and eight microsatellite markers. Temporal changes in the genetic composition were revealed by all three molecular markers, which may be attributed to seasonal changes in the NWS fly’s southern distribution. SNPs were employed for the first time for estimating the genetic structure in a NWS fly population; these results provide new clues and perspectives on its population genetic structure. This approach could have significant implications for the planning and implementation of management programs.

## 1. Introduction

Population genomics is an area of considerable growth, accompanying the development of next-generation sequencing (NGS) technologies, with the potential to improve population studies [1]. Many distinct approaches to obtain hundreds to thousands of molecular markers throughout the genome were developed in recent times [2,3]. One of them is genotyping-by-sequencing (GBS) [4,5], which is based on restriction enzymes to reduce genome representation and is capable of obtaining a huge number of single-nucleotide polymorphisms (SNPs) for several individuals at the same time. In the last years, several studies have been conducted using the GBS technique or similar methodologies for non-model insects [6,7,8,9,10,11,12].

This approach, which combines the sampling of many individuals and the characterization of a huge number of markers, allows the development of temporal studies, where samples from the same population are taken at different times with the aim to observe temporal changes in allelic frequencies, offering insights about the species population dynamics and demography. Effective population size (N_e_), an important demographic parameter usually difficult to accurately estimate, can be more robustly assessed with temporal sampling and population genomic data [13,14]. Most studies that aimed to infer demographic dynamics based on genetic data approached that based on historical timescales (i.e., thousands of generations) and not on an ecological timescale (i.e., tens of generations) [15]. In fact, the role of genetic variation on ecological differences must be considered when leading with both temporal and spatial allopatric populations [16]. Understanding demographic changes at an ecological timescale is particularly important for the New World screwworm (NWS) fly populations in its southern distribution (e.g., Uruguay), where the species is probably impacted by seasonal changes throughout the year.

The NWS fly, *Cochliomyia hominivorax* (Coquerel, 1858) (Diptera: Calliphoridae), is an ectoparasite that causes myiasis in warm-blooded vertebrates, including species of economic importance, consequently leading to negative economic impacts in livestock breeding [17]. In this species, females lay their eggs in wounds and natural host cavities. The emerged larvae feed on host living tissues and after three instars they fall to the ground to pupate, giving rise to adults after some days [17]. The species is intolerant to low temperatures and screwworms eggs fail to develop at temperatures lower than 12.3 °C [18]. The species is currently distributed over the Neotropical region, but its distribution originally extended from the Southern USA to Argentina [19]. This territory reduction involved systematic area-wide management programs based on the sterile insect technique (SIT) that eradicated the species from North America and continental Central America [20].

Several population genetic studies have been conducted in recent years in order to better understand the dynamics and distribution of the NWS fly (reviewed in [21]) with the aim to give basis for future implementation of similar control programs in its current area of occurrence. In South America, two genetic groups were identified based on different molecular markers, the North Amazon group (NAG) and the South Amazon group (SAG) [22,23]. Within the SAG, the results of some of the studies diverged about the finer scale in which genetic differentiation can be detected [24,25,26,27], which can be due to the evolutionary rate of the molecular markers investigated (mitochondrial DNA, microsatellites or *LcαE7* sequences). For this reason, the use of more informative molecular markers, such as SNPs obtained with the GBS methodology, could provide higher resolution in population genetic studies, allowing establishing boundaries (i.e., neighborhood size) and management units.

In the current study, we standardized the two-enzyme GBS protocol for the NWS fly and used SNPs markers to investigate temporal genetic changes in a Uruguayan NWS fly population at an ecological timescale. For a better understanding of the information provided by these markers we compared the obtained results with estimates based on mtDNA sequences and microsatellites for the same samples. Our study is the first to implement the GBS technique for this pest species, allowing its future implementation in spatial NWS fly population studies.

## 2. Material and Methods

### 2.1. Sampling Information

NWS fly samples were collected from wounds and natural cavities of sheep and cattle from the locality of Cañas (32°21′21.96″ S, 53°49′45.1″ W—Cerro Largo, Uruguay) in two distinct periods: February/March 2015 and March 2016, hereafter named as 2015-Sample and 2016-Sample, respectively. Genetic property was registered under SISGEN #A61EDD2. Larvae were collected from only one wound per animal to lower bias and were kept in absolute ethanol and freezer at −20 °C.

### 2.2. DNA Extraction

GBS libraries demand high quality DNA and the best quality total genomic DNA was obtained by extracting one to three larvae per wound using the CTAB method [28], with an additional step of RNAse treatment. Total DNA was eluted in 50 µL of AE buffer and stored at −20 °C. Extractions were quantified by fluorescence using the Qubit DNA quantification system (Invitrogen, Carlsbad, CA, USA), and their quality and purity were verified using a NanoDrop UV spectrophotometer (Techno Scientific, Wilmington, DE, USA). High quality DNA (260:280 ratio > 1.8, 230:260 ratio > 2.0) was obtained for 32 and 31 samples from 2015-Sample and 2016-Sample, respectively (a 45% extraction success). The DNA concentration per sample was normalized to 20 ng/µL for GBS libraries construction.

### 2.3. Single-Nucleotide Polymorphisms

SNPs were obtained using the genotyping-by-sequencing (GBS) approach. Two separated libraries were constructed with the individuals from 2015-Sample and 2016-Sample, based on standard protocols [4,5] with modifications described below. Total DNA was double-digested with two high fidelity restriction enzymes, *PstI–MspI* (New England Biolabs—NEB, Ipswich, MA, USA). Barcoded adapters were ligated on the rare cut site (i.e., *PstI* cut site) for each individual separately, and the common adapter was subsequently ligated on the common cut site (i.e., *MspI* cut site) for all individuals, using T4 DNA ligase (NEB). The samples were separated by year and pooled in two sets, which were amplified by multiplexed PCRs using standard forward primer A and modified reverse primer C with the Q5^®®^ high-fidelity DNA polymerase (NEB). PCR products were purified with the Agencourt Ampure XP beads (Beckman-Coulter, Inc., Brea, CA, USA), and the DNA amount was estimated with a NanoDrop UV spectrophotometer (Techno Scientific, Wilmington, DE, USA). Library quality was accessed with the evaluation of fragment sizes and the presence of adapter dimers in an Agilent BioAnalyzer 2100 at Life Sciences Core Facility (LaCTAD) from University of Campinas (UNICAMP). The two libraries were sequenced separately in an Illumina NextSeq 500 lane (Illumina, Inc. San Diego, CA, USA), using 150-bp single-end reads, at the IBTEC—UNESP (Botucatu, Brazil).

Stacks v1.48 de novo pipeline was used to demultiplex and perform SNP calling without a reference genome [29,30]. First, the *process_radtags* was used for raw data demultiplexing, removing low quality reads and with uncalled bases reads. Adaptors and restriction enzyme recognition sites were trimmed, and reads were filtered to consider only the initial 70 bp. We excluded 11 individuals after this process (five from 2015-Sample and six from 2016-Sample), due to the low number of reads (less than 1% from the total number of reads in the sequenced lane). This cleaned dataset was the input of *ustacks* function to assemble de novo loci present in each individual and to SNPs calling (applied parameters include minimum stack depth of 3 and allowed distance between stacks of 2 mismatches). Then, the data from each individual from both samples were merged into a catalog of loci using the *cstacks* function (allowed distance between catalog loci = 2), which contains all loci and alleles in the considered populations. Subsequently, each individual was matched against the catalog with *sstacks*. The function *populations* was executed for additional filtering considering (i) locus that must be present in both sampled years, (ii) only the first SNP of each locus, (iii) locus in at least 75% of individuals and (iv) a minimum minor allele frequency of 0.10.

Lastly, additional data filtering included pruning loci under Hardy–Weinberg disequilibrium and loci under selection from the two samples. Hardy–Weinberg tests were conducted in the web version of Genepop [31,32] considering a *p*-value of 0.05. Loci putatively under selection were detected with Lositan [33], which calculates the ratio between F_ST_ and expected heterozygosity (H_e_) values from each locus and compares them with the neutral distribution to detect outliers (i.e., loci under putative selective pressure) [34]. The program was run three times to lower bias [33]: (i) the first run considered all loci to calculate a neutral mean F_ST_, using 50,000 simulations, 99% confidence interval, infinite alleles mutation model and false discovery rate of 0.1%; (ii) the second run was conducted with the same parameters, except that only neutral loci from the first run were considered to recalculate the mean neutral F_ST_; (iii) the third run considered all loci again and the neutral F_ST_ calculated in the second run. Outlier loci in this last run were inferred as under selection and consequently excluded from the dataset used in population genetic analyses. These outlier loci sequences detected by Lositan were blasted, mapped and annotated against the National Center for Biotechnology Information Search database (NCBI) using Blast2Go [35] for gene ontology annotation. File conversions were conducted using PGDSpider v2.0.5.1 [36] for further population genetic analyses.

### 2.4. mtDNA

Three mitochondrial DNA fragments were amplified according to the procedures described in [23]: the B domain of the control region (CR) (ca. 541 bp) [37], the cytochrome c oxidase subunit I gene (COI) (ca. 892 bp) and the cytochrome c oxidase subunit II gene (COII) (ca. 686 bp). Purified PCR products were sequenced using the BigDye terminator cycle sequencing kit v3.0 (Applied Biosystems, Foster City, CA, USA) in an ABI 3730 automated DNA sequencer (Applied Biosystems, Foster City, CA, USA) at the Life Sciences Core Facility (LaCTAD) from State University of Campinas (UNICAMP).

CR, COI and COII sequences were independently aligned using ClustalX [38] and manually inspected in Mega v6 [39]. COI and COII fragments were translated to protein to confirm the open reading frame. Each insertion/deletion (*indel*) in the CR fragment was considered a single mutational step and recoded as a single position in the final alignment in Mega. Individual sequences of the three fragments were collapsed into haplotypes with Fabox (available at http://users-birc.au.dk/palle/php/fabox/index.php). Sequences of the three fragments for each individual were concatenated in a unique haplotype for further analyses.

### 2.5. Microsatellites

Eight polymorphic microsatellite loci were amplified and genotyped according to the procedures previously described [40,41]. PCR products were verified with 6% denaturing polyacrylamide gels stained with silver nitrate and the positive amplicons were multiplexed (4-plex, using different fluorophores (FAM, NED, PET and VIC)) for subsequent sequencing in an ABI 3500 genetic analyzer (Applied Biosystems). Each reaction received 0.4 μL of the molecular weight marker (Liz GeneScan 600 v2, Thermo Fisher Scientific), 9 μL of HI–DI formamide (Thermo Fisher Scientific) and up to 2 μL of the PCR products diluted in multiplex.

Microsatellite alleles were genotyped by size using GeneMarker v2.4.2 [42]. The presence and frequency of null alleles were estimated with Microchecker [43], which was also used to adjust allelic and genotypic frequencies.

### 2.6. Genetic Diversity and Population Differentiation

Genetic diversity and population structure analyses were conducted for each molecular marker in order to detect temporal changes in the NWS fly population from Cañas.

Filtered neutral SNPs were used to investigate nucleotide diversity. Observed heterozygosity (H_o_), expected heterozygosity (H_e_) and Wright’s F-statistic (F_IS_) were estimated with the function *populations* available in the Stacks pipeline. Genetic differentiation between 2015-Sample and 2016-Sample was estimated with a nonhierarchical AMOVA with Arlequin v3.5 [44], with the Bayesian approach implemented in Structure v2.3.3 [45] and with a discriminant analysis of principal components (DAPC) [46]. AMOVA significance was obtained by 10,000 permutations. The parameters for the Structure analysis included admixture model with correlated allele frequencies, 100,000 MCMC replications, 10,000 burn-in, K varying from 1 to 5 and 10 iterations for each K value. Evanno et al. method [47] was used to determine the best K value using the application Structure Harvester v0.6.94 [48]. Results from this best K value were summarized with Clumpp v1.1.2 [49] and represented with Distruct v1.1 [50]. The DAPC analysis was conducted with the R package adegenet [51]. The function *find.clusters* was used to determine genetic clusters, based on principal component analysis (PCA) retaining the maximum number of PCs and the Bayesian information criterion (BIC) to generate a graph of optimum K. This procedure produced a scatterplot and a smaller number of PCs were retained then, following recommendations in the package. Subsequently, the contributions of the alleles to the best clustering scenario was also computed, considering a threshold of 0.005.

Mitochondrial DNA haplotype diversity (Ĥ), nucleotide diversity (π) and overall genetic structure (nonhierarchical AMOVA) were estimated using Arlequin v3.5 [44] with statistical significance obtained with 10,000 permutations. A parsimony haplotype network was constructed with PopART v1.7 [52] using the TCS method with 95% connection limit [53] to recover relationships among concatenated sequences.

The genetic variability of microsatellite loci was accessed through allelic richness (AR) with MSA v4.05 [54]; observed (H_o_) and expected (H_e_) heterozygosities with GenAlex v6.5 [55]; and estimates of F_IS_ with the web version of Genepop [31,32]. Each locus was tested for Hardy–Weinberg equilibrium with the web version of Genepop [31,32], considering the Markov chain model from [56] and using the following parameters: 1000 series, 1000 iterations and 1000 steps of “dememorization”. Linkage disequilibrium between pairs of loci within each sample was investigated using the web version of Genepop [31,32]. Genetic structure was estimated by a nonhierarchical AMOVA with Arlequin v3.5 [44], using 10,000 permutations to access statistical significance and by a Bayesian clustering analyses with Structure 2.3.3 [45], using the same parameters described above.

### 2.7. Demographic Inferences

For both SNPs and microsatellite data, two-sample effective population size (N_e_) estimates were calculated using Jorde and Ryman method [57] in NeEstimator v2.01 [58]. This analysis considered a minimum allele frequency (MAF) cutoff of 0.01, 0.02 and 0.05 and 18 generations/year (considering that the NWS fly generation time is approximately 21 days under laboratory conditions at 25 °C [17]).

The demographic history of the NWS fly samples was inferred from mitochondrial data with Tajima’s D [59] and Fu’s Fs [60] neutrality tests and mismatch distribution analysis using Arlequin v3.5 [44]. For both summary statistics, values near zero are indicative of population size stability, negative values are indicative of recent population expansion and positive values are indicative of population bottlenecks [59,60]. Mismatch distribution analysis was performed with 10,000 permutations for statistical significance. The *Raggedness* index (*rg*) was used to measure the adjustment to a population expansion model.

## 3. Results

### 3.1. Single-Nucleotide Polymorphisms

Sequencing of both 2015 and 2016 GBS libraries yielded more than 135 Gb of raw data (Table 1). After the initial filtering steps, 89% and 79% of these raw sequences were retained for Stacks pipeline downstream steps for 2015-Sample and 2016-Sample, respectively (Table 1).

SNP calling after successive filtering recovered 1137 loci in 52 individuals (11 individuals were filtered from the total 63 due to the low number of reads, resulting in a final dataset of 27 and 25 individuals in 2015 and 2016 Samples, respectively). From the 1137 loci 52 were in Hardy–Weinberg disequilibrium and 257 were detected as outliers (i.e., putatively under selection). From these 257 outliers’ loci, only 50 were successfully annotated against NCBI database and were related to 171 Gene Ontologies (GOs); no information was found for the remaining 207 outlier loci. Most of the annotated sequences were associated with metabolic process (72.22%) when considering the category of biologic process, such as proteolysis (12%), regulation of transcription by RNA polymerase II (10%), positive regulation of gene expression (8%) and negative regulation of transcription (7%) (Figure S1). A small percentage (1%) of sequences has GOs associated with phosphate-containing compound metabolic process. Specifically, the locus 20,670 can be related to organophosphate metabolic process via adenylate cyclase type 2 (Table S1), showing high similarity to sequences from *Musca domestica* (XP_011296241.1) and *Stomoxys calcitrans* (XP_013113360.1) (identities 21/22, 95%). The locus 10,178 blasted with the highest similarity to *Lucilia cuprina* cadherin-related tumor suppressor (KNC26434.1) (identity 22/23, 96%), which is known to be related to *Bt*-resistance in insects [61,62]. The locus 226,347 was annotated as glutathione S-transferase, with high similarity to sequences from different Diptera species, as *Sarcophaga dux* (QGJ03700.1) (identity 22/23, 96%), *Bactrocera oleae* (XP_014086721.1) (identity 23/23, 100%), *Ceratitis capitata* (XP_004523110.1) (identity 22/23, 96%), *Musca domestica* (5ZWP_A) (identity 22/23, 96%) and *Lucilia cuprina* (P42860.2) (identity 22/23, 96%). Glutathione transferase is highly related to insecticide resistance in insects [63,64].

After all the filtering steps described above, the data matrix for downstream population analyses was represented by 828 neutral loci. Mean expected heterozygosities for both 2015- and 2016-Samples were similar, being somewhat lower in 2016-Sample (0.1317 for 2015-Sample and 0.1141 for 2016-Sample) and were slightly higher than the mean observed heterozygosities (0.1113 for 2015-Sample and 0.1044 for 2016-Sample), culminating in small positive F_IS_ estimates (0.0981 for 2015-Sample and 0.0498 for 2016-Sample). Besides the 52 loci with deviations from Hardy–Weinberg equilibrium in both samples (previously excluded), 116 and 94 loci showed deviations in 2015- and 2016-Samples, respectively (data not shown).

**Figure 1:**
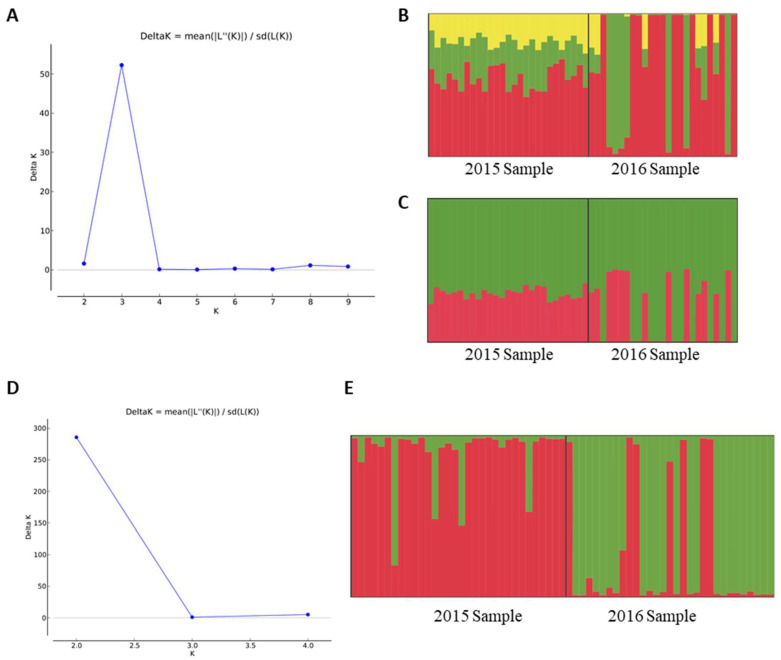
Structure results based on single-nucleotide polymorphisms (SNPs) and microsatellite data. (**A**) DeltaK from Evanno et al. method [47] indicating three clusters as the best K value for SNPs; (**B**) Structure inference based on SNPs, considering K = 3; (**C**) Structure inference based on SNPs, considering K = 2; (**D**) DeltaK from Evanno et al. method [47] indicating two clusters as the best K value for microsatellites; (**E**) Structure inference based on microsatellites considering K = 2. In (**B**,**C**,**E**), each color represents a genetic group and each vertical line represents an individual (for SNPs there is a total of 52 individuals, while for microsatellites there are 63). Each individual is colored proportionally to its probability of assignment to each genetic group.

**Figure 2:**
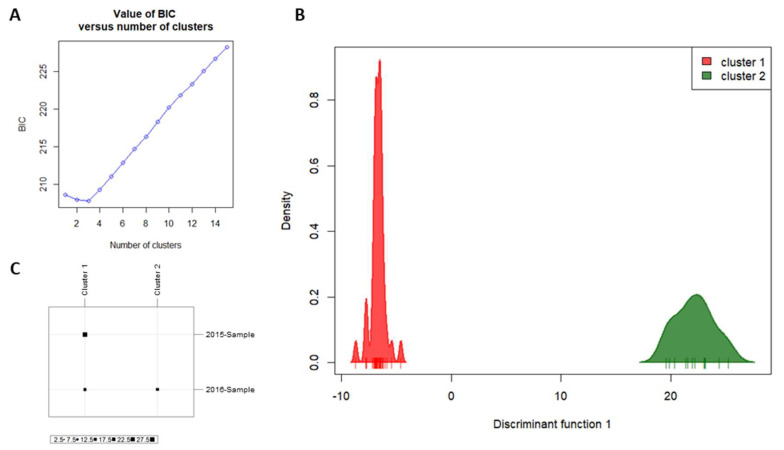
Discriminant analysis of principal components (DAPC) results based on SNP data. (**A**) Graph of Bayesian information criterion (BIC) values versus number of clusters. The lowest BIC value indicates K = 3 as the optimal number of clusters. However, we considered K = 2 as the best option to explain the distribution of variation in temporal samples of New World screwworm (NWS) flies; (**B**) density plot of the two clusters; (**C**) graph indicating the population of origin of the individuals from each cluster. Cluster 1 is composed of 27 individuals from 2015-Sample and 13 individuals from 2016-Sample, while cluster 2 has only individuals from 2016-Sample (12 individuals).

AMOVA based on neutral SNPs indicated that populations were only subtly genetically differentiated (F_ST_ = 0.01444, *p* < 0.05). Bayesian inferences of population structure based on neutral SNPs also indicated some population differentiation over time (Figure 1A–C). Delta K from Evanno et al. method [47] indicated K = 3 as the best clustering scenario (Figure 1A), but one of the clusters (presented in yellow) is apparently misleading, since no individuals have a high admixture proportion assigned to it (Figure 1B). Thus, we considered that K = 2 seems to be more plausible since we are dealing with two temporal samplings (Figure 1C). Similarly, the BIC analysis from DAPC resulted in the smallest value with K = 3 (Figure 2A). However, the choice of K is not always clear, considering that clustering programs assign individuals to groups in order to create a caricature of a complex reality [65]. Therefore, although obtaining the smallest value of BIC with K = 3 (results from DAPC analysis considering K = 3 were presented in Figure S2), we considered K = 2 as the best option to explain the distribution of variation in temporal samples of NWS flies (Figure 2B), since the reduction in BIC values between K = 3 and K = 2 was very small. The two clusters when considering K = 2 were mainly determined by 13 loci (Figure S3). Cluster 1 mainly comprised individuals from 2015-Sample, while cluster 2 exclusively comprised individuals from 2016-Sample (Figure 2C). Similar results for DAPC analysis were found when considering the 257 loci putatively under selection combined with the 828 neutral loci (Figure S4), indicating that population genetic structure is not due to selection.

### 3.2. mtDNA

Concatenated mtDNA sequences resulted in 21 haplotypes for the 63 individuals, from which 13 were unique. The most frequent haplotypes H1 (32/63) and H2 (5/63) are shared between 2015- and 2016-Samples (Table 2). The 2015-Sample is the most diverse in number of haplotypes and both 2015- and 2016-Samples showed high and moderate haplotype diversity estimated by Ĥ, respectively and low nucleotide diversity (π) (Table 2).

**Figure 3:**
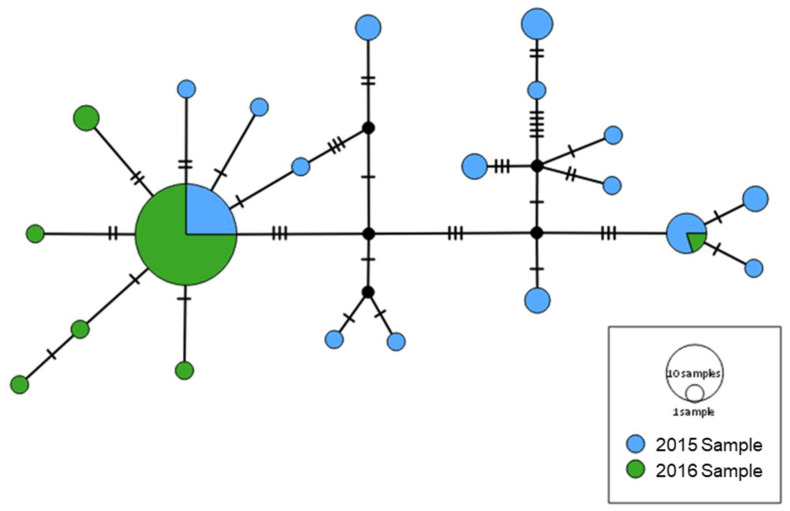
Haplotype network from mtDNA concatenated sequences of control region (CR), cytochrome c oxidase subunit I gene (COI) and cytochrome c oxidase subunit II gene (COII). Circles sizes are proportional to the number of sequences representing the haplotype.

Haplotype network based on mtDNA concatenated sequences indicated that 2015- and 2016-Samples differ in haplotypes composition and frequency, although they share the two most frequent haplotypes (Figure 3). Additionally, the 2016-Sample show a star-shaped pattern of haplotype variation which is considered indicative of population expansion.

AMOVA based on mtDNA data revealed genetic structure between 2015- and 2016-Samples (F_ST_ = 0.27537, *p* < 0.001), which indicates that the NWS fly population from Cañas changed considerably from one year to another when the mitochondrial information is considered.

### 3.3. Microsatellites

Microsatellite loci showed a moderate to high degree of variability, with H_o_ ranging from 0.355 to 0.833 and H_e_ from 0.361 to 0.852 (Table 3). No locus showed significant deviations from Hardy–Weinberg equilibrium after Bonferroni correction (*p* < 0.006) in the 2015-Sample. Otherwise, four out of eight loci significantly deviated from Hardy–Weinberg equilibrium in 2016-Sample after correction (*p* < 0.006), with CH15 and CH26 exhibiting heterozygote deficit (i.e., positive F_IS_) and CH05 and CH14 exhibiting excess of heterozygotes (i.e., negative F_IS_).

Tests for detecting genotypic linkage disequilibrium (LD) found significant association between pairs of loci only in the 2016-Sample, with 12 cases for *p* < 0.05 and 8 cases after Bonferroni correction (*p* < 0.006) (Table S2). AMOVA for microsatellites indicated that populations were subtly differentiated (F_ST_ = 0.08385, *p* < 0.001). Bayesian inferences of population structure for microsatellite data also indicate population differentiation over time, revealing the presence of two genetic clusters (Figure 1D,E).

### 3.4. Demographic Inferences

Estimates of effective population size (N_e_) based on SNPs and microsatellite data diverged about 3.5-fold: estimates based on SNPs indicated around 250 individuals, while those based on microsatellites indicated a N_e_ of approximately 70 individuals (Table 4).

Neutrality tests based on mtDNA were statistically significant only in one case: Tajima’s D for 2016-Sample (Table 5). The negative value of Tajima’s D is indicative of population expansion. Since only 2016-Sample revealed some signal of population size change, the mismatch distribution analysis was conducted with this population alone (Figure S5). This analysis resulted in a statistically insignificant *Raggedness* index (*rg* = 0.2978, *p* = 1.00), indicating that we cannot reject the null hypothesis of population expansion.

## 4. Discussion

Single-nucleotide polymorphisms were hereby obtained for the NWS fly (*C. hominivorax*) using the two-enzyme based genotyping-by-sequencing (GBS) libraries for the first time. The GBS technique [4,5] radically changed the state of the art of population genomics, allowing the simultaneous obtainment of many informative genomic markers of any species for several individuals, at a relatively low cost [2]. This population genomics approach allowed a refinement in population and demographic parameters inference, which otherwise would not be obtained using most regularly employed markers, such as mtDNA sequencing and microsatellites. SNPs are believed to be superior to microsatellites for elucidating historical demography, since they represent an unbiased sampling of genomic variation [66]. Therefore, we can consider that N_e_ estimates based on SNP data (250 individuals ranging from 211.2 to 287.8) can be more accurate for our NWS fly sampling than microsatellite estimates (70 individuals ranging from 39.5 to 100.5). In spite of that, these results should be considered with caution, since accurate estimation of N_e_ and population demographic changes in a contemporary time scale (20 generations) is dependent on the number of samples and the targeted number of RADseq (or GBS) loci (i.e., very large SNP data sets, with more than 25,000 SNPs, are necessary to detect population declines) [14], as well as dependent on the proportion of missing data and minor allele frequency (MAF) [67]. Our N_e_ estimates of approximately 70 and 250 individuals for microsatellite and SNP loci, respectively, is low compared to other insects. Estimates of N_e_ for *Aedes aegypti* across 17 geographic localities and time points resulted in values slightly different from the ones we found, considering the same method of Jorde and Ryman [57]: for microsatellite loci, estimated N_e_ averaged 303.3, ranging from 25.0 to 1181.0, while for SNPs the average was 166.0, ranging from 22.9 to 549.2 [68].

Wounded-host availability, extensive insecticide use by farmers, seasonality and climate are important factors affecting yearly population size of the NWS fly in its southernmost distribution. We consider two likely hypothesis for NWS fly populations dynamics in Uruguay: (i) during winter unfavorable conditions, characterized by cold temperatures and low precipitation, the species migrates to northern latitudes and recolonize the southern region when climate became favorable; alternatively (ii) during the winter the NWS fly populations are reduced, surviving in local refugees like riverine forests and when climate became favorable the populations expand. This cyclic phenomenon of population size changes based on annual seasons is well known for many insect species [69]. However, we were unable to formally test both hypothesis (i.e., migration–recolonization, maintenance–recolonization) due to the absence of samples for additional years and specially samples taken at the beginning and the end of the favorable season.

The three investigated molecular markers, SNPs, mtDNA and microsatellites, indicated temporal genetic structure between two consecutive years for the NWS fly populations from Cañas, Uruguay. However, SNP data variability showed no drastic change from one year to the next: a similar number of loci showed deviations from Hardy–Weinberg equilibrium and mean observed and expected heterozygosity were similar. Besides that, population structure analyses indicated differentiation between years. SNPs are bi-allelic and expected to be less variable than microsatellites [13]. Despite of this, many studies reported sufficient power for SNPs to detect population structure, even in cases where divergence between populations is very low (e.g., [70]). We indeed found small genetic structure between 2015- and 2016-Sample based on SNPs and microsatellites, whereas mtDNA indicates higher structure. That difference suggests differential permanence of males and females from one year to the next in our population, in contrast with data on dispersion of NWS flies [71]. Additionally, random variation due to small population size may result in genetic structure between populations. In fact, populations of NWS flies are constantly controlled at field conditions to diminish their populations. As result, the next year population will be composed by individuals that survived during the anterior year with the addition of individuals that may migrate from neighbor populations.

Although we have not found any important contribution of loci under selection for our NWS fly samples genetic structure, we were still able to annotate some of them as associated with insecticide resistance, which is of interest since it represents a challenge to species control. One of these genes is related to organophosphate metabolic process, considered the main insecticide resistance mechanism for *C. hominivorax* [72,73]. Another locus is related to *Bt*-resistance in insects and, despite not being common for *C. hominivorax* control, it was demonstrated that *Bacillus thuringiensis* (*Bt*) isolates have applicability for Calliphoridae species control, including screwworms (Patent US6056953A—Hickle et al. 2000). Possibly, our NWS samples were exposed to *Bt* in the field, since it is widely used for controlling sympatric herbivorous species, both as transgenic part of the genome of host plants and as sprays [74,75]. We also found a glutathione transferase sequence, a diverse gene family highly associated with insecticide resistance in insects [63,64]. Annotation is fundamental to identify genomic regions under selection since it adds the adaptive process layer to analyses in population genomics studies, leading to a better understand of population structure patterns [76]. Accordingly, loci putatively under selection, many of them annotated as resistance to insecticides functions, are suggested to be the main factors responsible for the genetic structure found in *Spodoptera frugiperda* (Lepidoptera, Noctuidae) Brazilian populations [10]. Neither mtDNA nor microsatellites could provide such refined information, and future studies with SNPs in NWS fly populations should confirm the potential role of loci under selection in the spatial population genetic structure.

Despite limitations due to the low number of samples, SNPs obtained by GBS from *C. hominivorax* samples demonstrate to be a potential powerful marker for both spatial and temporal population genomic studies for this species. Additionally, these markers allow the detection of loci putatively under selection that can be associated with insecticide resistance and other important functions for insect pest control.

## Figures and Tables

**Table 1:** Sequencing results from 2015-Sample and 2016-Sample GBS libraries.

	2015-Sample	2016-Sample
Total number of sequences	145,692,719	136,389,952
Reads containing adapter sequence	9,343,260	6,878,774
Ambiguous barcodes	934,983	13,656,605
Low quality reads	51,524	164,258
Ambiguous tags	5,134,707	7,798,267
Retained reads	130,228,245 (**89%**)	107,892,048 (**79%**)

**Table 2:** Mitochondrial DNA genetic diversity. N—number of individuals; Nh—number of haplotypes; Ĥ—haplotype diversity; π—nucleotide diversity.

Sample	N	Nh	Haplotypes (# Individuals)	Ĥ (SD)	π (SD)
**2015**	32	19	H1 (8), H2 (4), H3 (2), H4 (2), H5 (2), H6 (3), H8 (2), H9 to H17 (1)	0.9375 (0.0286)	0.004530 (0.002372)
**2016**	31	8	H1 (24), H2 (1), H7 (2), H18 to H21 (1)	0.4538 (0.1109)	0.001858 (0.001064)

**Table 3:** Microsatellite genetic diversity for each locus in each NWS fly population. N—sample size; Na—number of alleles per locus; AR—allelic richness; H_o_—observed heterozygosity; H_e_—expected heterozygosity; F_IS_—inbreeding coefficient. Significant departures from Hardy–Weinberg equilibrium were indicated by an asterisk (*) in the value of H_e_ (*p* < 0.006).

Sample		Loci							
		CH01	CH05	CH09	CH12	CH14	CH15	CH24	CH26
**2015-Sample**	**N**	23	32	31	30	24	21	21	32
**(N = 32)**	**Na (AR)**	7 (7)	5 (4.97)	5 (5)	11 (11)	4 (4)	8 (7.98)	6 (6)	11 (10.75)
	**H_o_**	0.522	0.656	0.355	0.833	0.375	0.667	0.714	0.781
	**H_e_**	0.693	0.665	0.361	0.804	0.533	0.811	0.787	0.815
	**F_IS_**	0.2677	0.0291	0.0337	−0.0197	0.3157	0.2011	0.1163	0.0572
**2016-Sample**	**N**	31	31	31	31	31	19	31	30
**(N = 31)**	**Na (AR)**	6 (5.99)	6 (6)	5 (5)	7 (6.97)	5 (4.90)	5 (5)	4 (4)	9 (9)
	**H_o_**	0.548	0.645	0.613	0.710	0.645	0.579	0.613	0.833
	**H_e_**	0.677	0.583 *	0.624	0.729	0.616 *	0.778 *	0.670	0.852 *
	**F_IS_**	0.2056	−0.0909	0.0339	0.0435	−0.0318	0.2813	0.1009	0.0391

**Table 4:** N_e_ estimates based on SNP and microsatellite (SSR) data (Jorde and Ryman method [57]).

		Lowest Allele Frequency Used			
		0.05	0.02	0.01	0+
**Harmonic Mean Sample Size**		27.1	27	27.2	27.5
**SNP**	**Fs**	0.07969	0.08043	0.07811	0.07739
	**F′**	0.03631	0.03710	0.03469	0.03435
	**N_e_** **(95% CI, parametric)**	247.9 (211.4–287.2)	242.6 (211.2–276.1)	259.5 (233.3–287.0)	262.0 (237.3–287.8)
**SSR**	**Fs**	0.17722	0.17167	0.17052	0.17003
	**F′**	0.13531	0.13013	0.12905	0.12859
	**N_e_** **(95% CI, parametric)**	66.5 (39.5–100.5)	69.2 (43.9–100.1)	69.7 (45.1–99.6)	70.0 (46.5–98.2)

**Table 5:** Neutrality tests for mtDNA and their respective *p*-values (* indicates significant *p*-value).

	Tajima’s D	*p*-Value	Fu’s FS	*p*-Value
**2015-Sample**	−0.32833	0.416	−2.77043	0.16600
**2016-Sample**	−2.38060	<0.001 *	1.10613	0.72700

## Data Availability

GBS raw data can be accessed at NCBI SRA Bioproject PRJNA657064 and Biosamples SAMN15814514, SAMN15814515. Mitochondrial DNA sequences were deposited in the GenBank database under the accession numbers: MT875913 - MT875975 for COI, MT875976 - MT876038 for COII and MT876039 - MT876101 for CR.

## Supplementary Materials

The following are available online at https://www.mdpi.com/2075-4450/11/8/539/s1, Figure S1: Pie charts of multilevel gene ontology annotation (GO) of loci putatively under selection in each category (cellular component, molecular function and biologic process), Figure S2: DAPC results based on SNP data, Figure S3: A) Contributions of the alleles to the clustering scenario of K = 2 and B) allele frequencies of the 13 loci that are above the threshold of 0.005 in each of the two clusters, Figure S4: DAPC results based on 1085 SNP data (828 neutral loci + 257 outlier loci detected by Lositan), Figure S5: Mismatch distribution analysis for mtDNA sequences from 2016-Sample, Table S1: Gene ontology annotation (GO) of loci putatively under selection, Table S2: Pairwise linkage disequilibrium between microsatellite loci.

## Additional Information

The manuscript is part of an unpublished dissertation from L.W.B. at the University of Campinas (Brazil).
