# Supplementary material for: Genetic Differentiation of a New World Screwworm Fly Population from Uruguay Detected by SNPs, Mitochondrial DNA and Microsatellites in Two Consecutive Years
Source: Insects. 2020 Aug 16;11(8):539. doi: 10.3390/insects11080539 (PMC7469150; doi:10.3390/insects11080539)
Supplement: Supplementary file 1 [file insects-11-00539-s001.pdf]

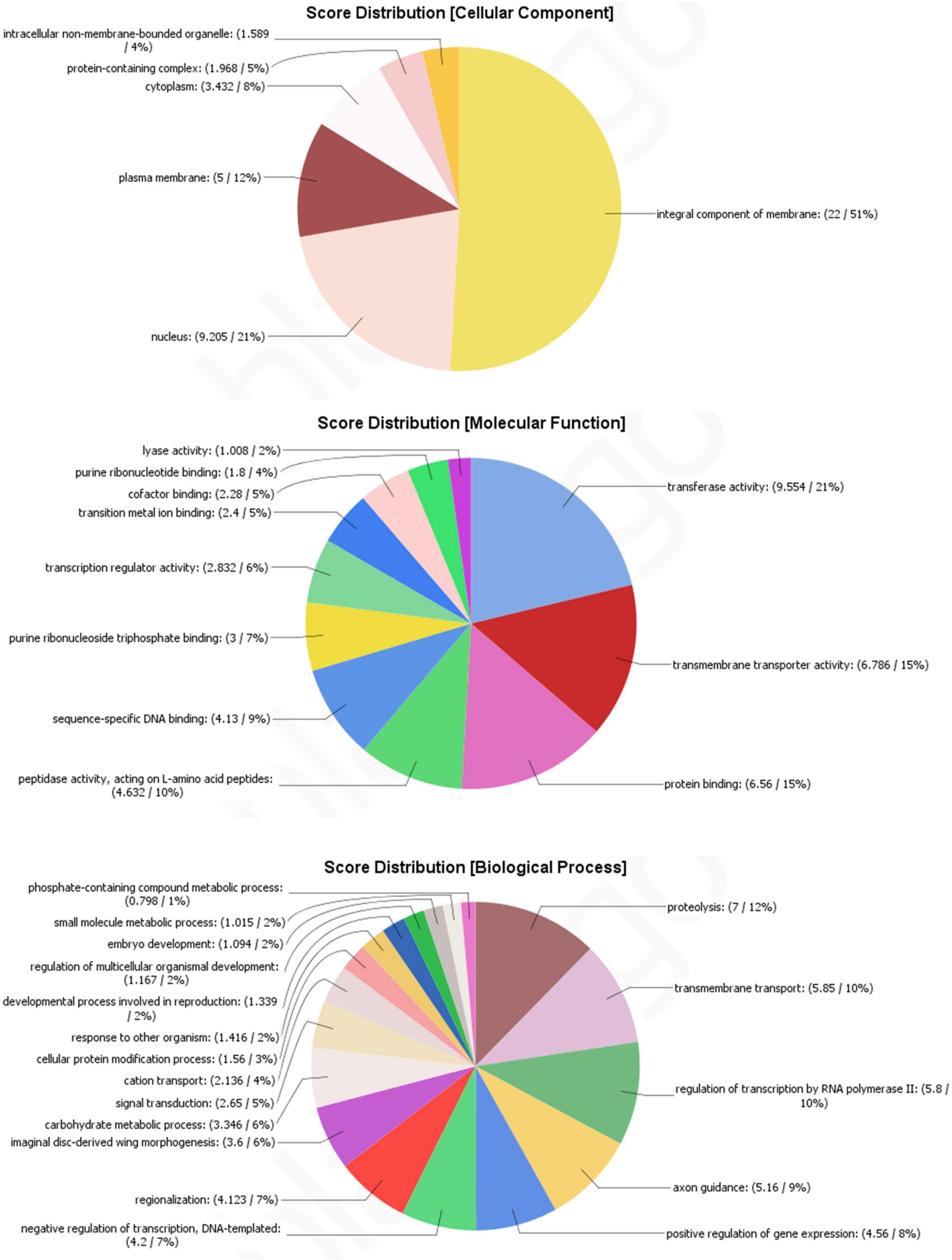

Supplementary Figure S1. Pie charts of multilevel Gene Ontology annotation (GO) of loci putatively under selection in each category (Cellular Component, Molecular Function and Biological Process).

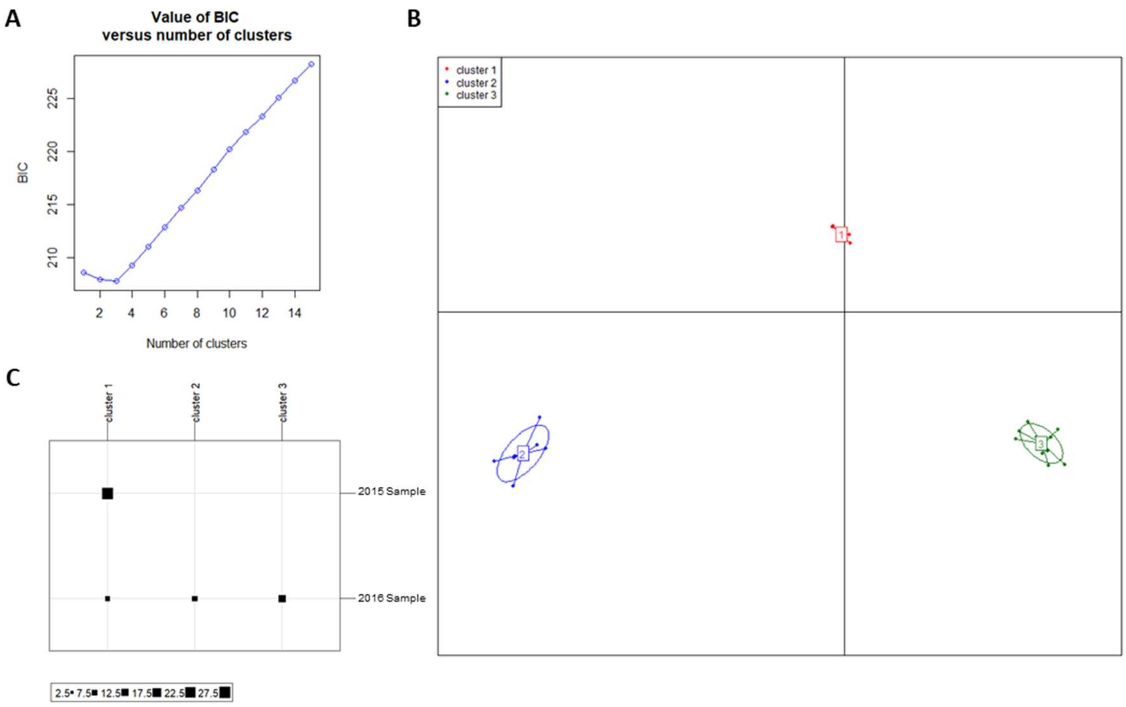

Supplementary Figure S2. DAPC results based on SNP data. A) Graph of BIC values versus number of clusters. The lowest BIC value indicates K=3 as the optimal number of clusters; B) Scatterplots for spatial visualization of the three clusters; C) Graph indicating the population of origin of the individuals from each cluster. Cluster 1 is composed of 27 individuals from 2015-Sample and 6 individuals from 2016-Sample, while clusters 2 and 3 have only individuals from 2016-Sample (7 and 12 individuals, respectively).

A

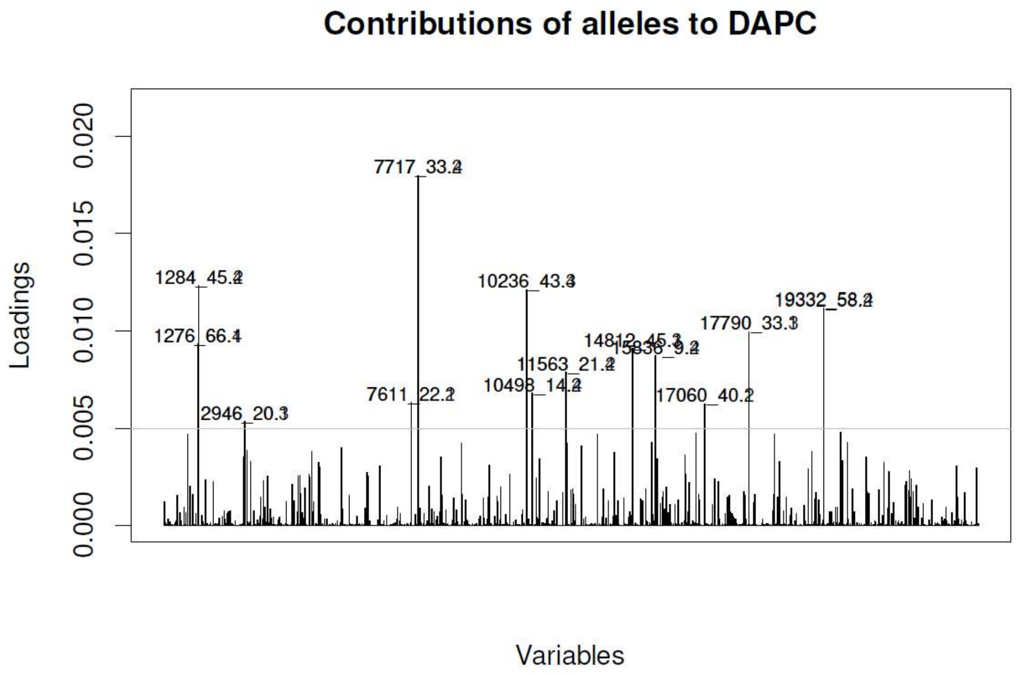

Contributions of alleles to DAPC
Loadings
0.000
0.005
0.010
0.015
0.020
Variables
7717_33.2
1284_45.2
1276_66.1
2946_20.3
7611_22.2
10236_43.3
10498_14.2
11563_21.2
14812_45.3
15836_9.2
17060_40.2
17790_33.3
19332_58.2

B

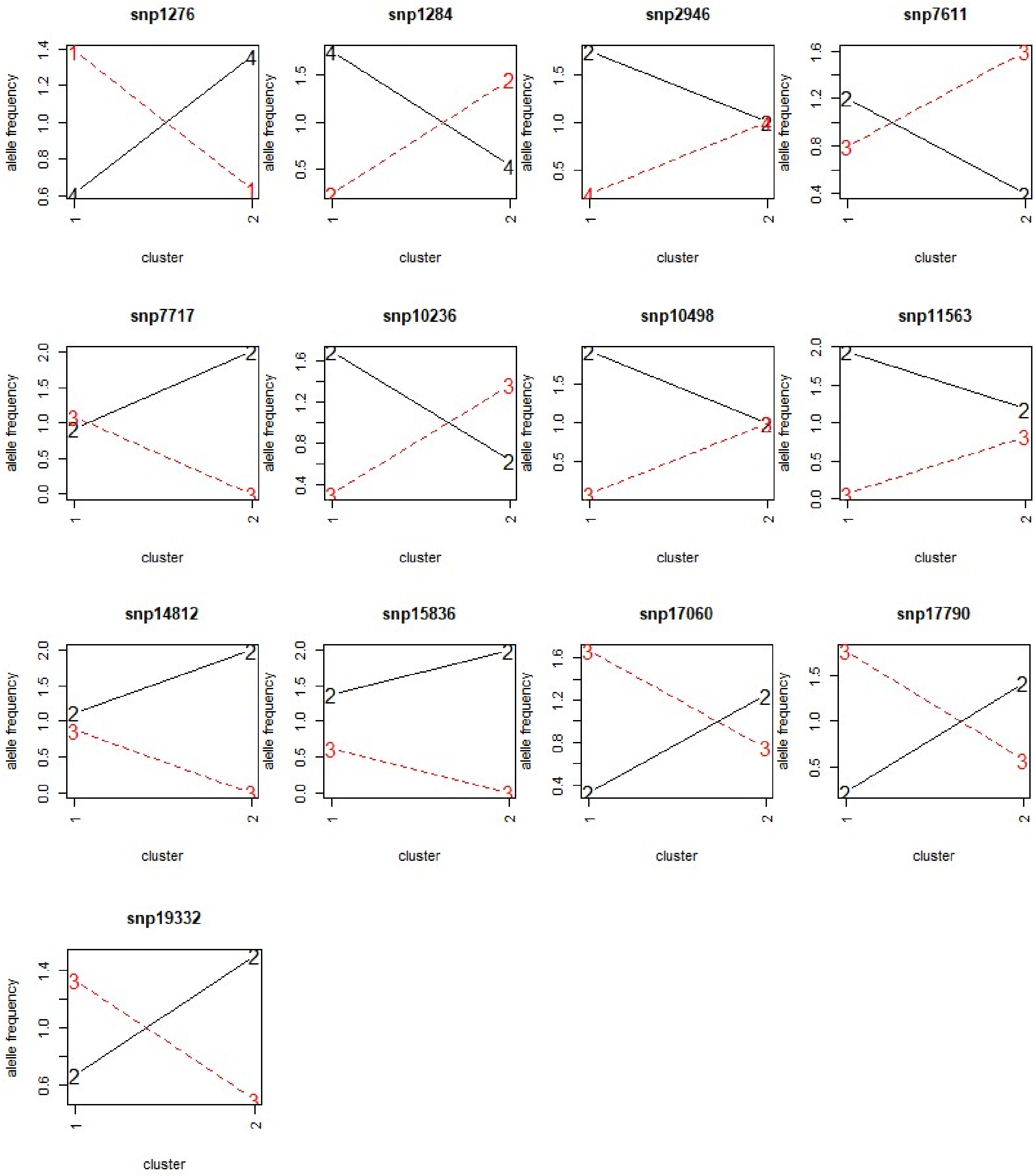

Supplementary Figure S3. A) Contributions of the alleles to the clustering scenario of K=2 and **B)** the allele frequencies of the 13 loci that are above the threshold of 0.005 in each of the two clusters.

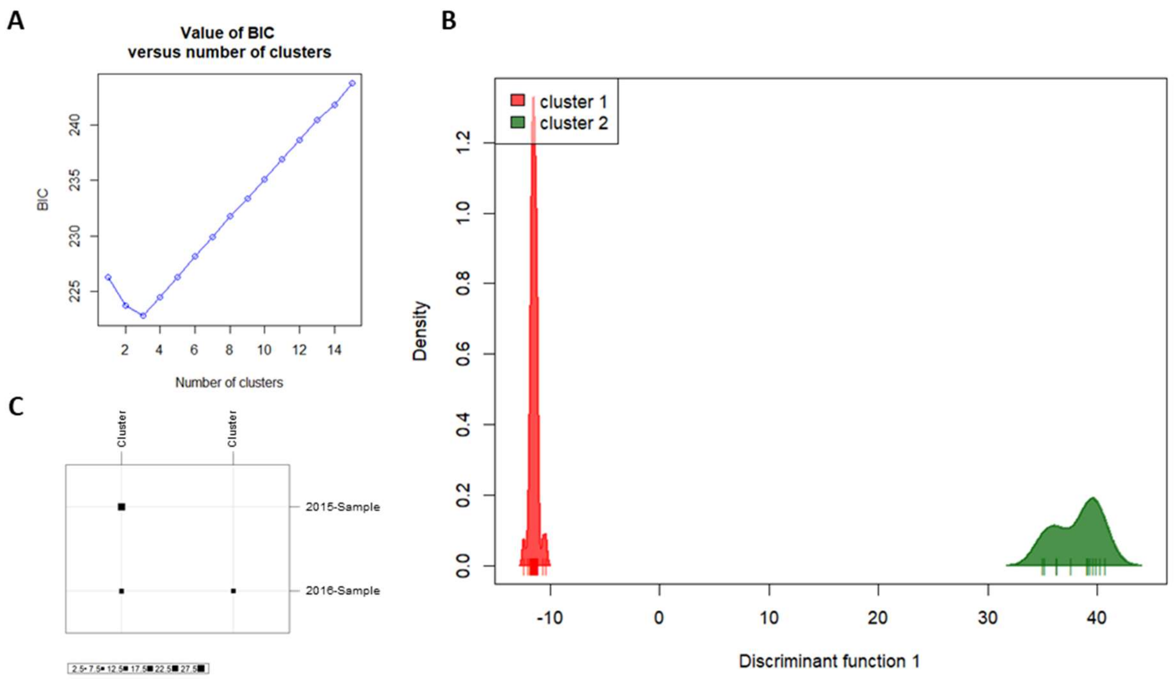

Supplementary Figure S4. DAPC results based on 1085 SNP data (828 neutral loci + 257 outlier loci detected by Lositan). A) Graph of BIC values versus number of clusters. The lowest BIC value indicates K=3 as the optimal number of clusters. However, we considered K = 2 as the best option to explain the distribution of variation in temoral samples of NWS flies; B) Density plot of the two clusters; C) Graph indicating the population of origin of the individuals from each cluster. Cluster 1 is composed of 27 individuals from 2015-Sample and 13 individuals from 2016-Sample, while cluster 2 has only individuals from 2016-Sample (12 individuals).

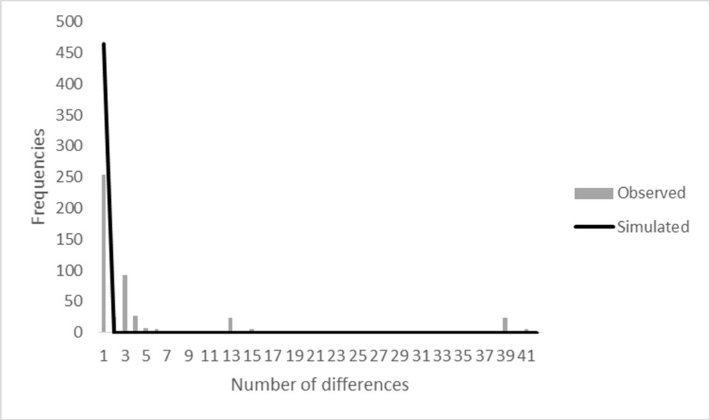

Supplementary Figure S5. Mismatch distribution analysis for mtDNA sequences from 2016 Sample. This analysis resulted in a non-significant *Raggedness* index (rg = 0.2978, P = 1.00), indicating that we cannot reject the null hypothesis of population expansion.

Supplementary Table S1. Gene Ontology annotation (GO) of loci putatively under selection. Locus Name is the catalog locus identifier generated by the *cstacks* function from the Stacks pipeline. F = Molecular Function, P = Biological Process, C = Cellular Component

Locus Name	Description	GO IDs	GO Names
1146	hypothetical protein FF38_05889	F:GO:0003676; C:GO:0016021; F:GO:0016787; F:GO:0046872	F:nucleic acid binding; C:integral component of membrane; F:hydrolase activity; F:metal ion binding
2080	gamma-glutamyltranspeptidase 1 isoform X2	F:GO:0005272; P:GO:0006508; P:GO:0006751; C:GO:0016021; P:GO:0035725; F:GO:0036374	F:sodium channel activity; P:proteolysis; P:glutathione catabolic process; C:integral component of membrane; P:sodium ion transmembrane transport; F:glutathione hydrolase activity
2202	cathepsin L1 isoform X2	P:GO:0006508; F:GO:0008234	P:proteolysis; F:cysteine-type peptidase activity
3714	Phenylalanyl-tRNA synthetase subunit beta, related	C:GO:0016021	C:integral component of membrane
4792	rab2 gtpase, putative	F:GO:0003924; F:GO:0004672; F:GO:0005506; F:GO:0005524; F:GO:0005525; P:GO:0006468; F:GO:0009055; C:GO:0016021; F:GO:0016705; F:GO:0020037; P:GO:0022900; F:GO:0031418	F:GTPase activity; F:protein kinase activity; F:iron ion binding; F:ATP binding; F:GTP binding; P:protein phosphorylation; F:electron transfer activity; C:integral component of membrane; F:oxidoreductase activity, acting on paired donors, with incorporation or reduction of molecular oxygen; F:heme binding; P:electron transport chain; F:L-ascorbic acid binding
4862	facilitated trehalose transporter Tret1-2 homolog	C:GO:0016021; F:GO:0022857; P:GO:0055085	C:integral component of membrane; F:transmembrane transporter activity; P:transmembrane transport
5494	zinc finger protein with KRAB and SCAN domains 5-like	F:GO:0003676	F:nucleic acid binding
5769	serine/threonine protein kinase	F:GO:0004674; F:GO:0005524; C:GO:0005814; P:GO:0006468; P:GO:0007099	F:protein serine/threonine kinase activity; F:ATP binding; C:centriole; P:protein phosphorylation; P:centriole replication
5876	ecdysone-induced protein 75B, isoforms C/D	P:GO:0000122; F:GO:0003707; F:GO:0004879; C:GO:0005634; P:GO:0007553; F:GO:0008270; P:GO:0018990; P:GO:0019730;	P:negative regulation of transcription by RNA polymerase II; F:steroid hormone receptor activity; F:nuclear receptor activity; C:nucleus; P:regulation of ecdysteroid metabolic process; F:zinc ion binding; P:ecdysis, chitin-based cuticle; P:antimicrobial humoral response;

		F:GO:0020037;<br>P:GO:0030522;<br>P:GO:0035075;<br>P:GO:0043401;<br>F:GO:0043565;<br>P:GO:0048477	F:heme binding;<br>P:intracellular receptor signaling pathway;<br>P:response to ecdysone;<br>P:steroid hormone mediated signaling pathway;<br>F:sequence-specific DNA binding;<br>P:oogenesis
6017	hypothetical protein FF38_08830	C:GO:0016021	C:integral component of membrane
7240	30S ribosomal protein S6	F:GO:0003735;<br>C:GO:0005840;<br>P:GO:0006412;<br>F:GO:0019843	F:structural constituent of ribosome;<br>C:ribosome;<br>P:translation;<br>F:rRNA binding
8297	glutaconyl-CoA decarboxylase subunit alpha	F:GO:0016874;<br>F:GO:0018801	F:ligase activity;<br>F:glutaconyl-CoA decarboxylase activity
8312	CG7777, isoform D	F:GO:0015267;<br>C:GO:0016021;<br>P:GO:0055085	F:channel activity;<br>C:integral component of membrane;<br>P:transmembrane transport
8535	hypothetical protein FF38_00347	F:GO:0004930;<br>P:GO:0007186;<br>C:GO:0016021	F:G protein-coupled receptor activity;<br>P:G protein-coupled receptor signaling pathway;<br>C:integral component of membrane
8914	hypothetical protein FF38_12612	F:GO:0004222;<br>P:GO:0006508	F:metalloendopeptidase activity;<br>P:proteolysis
9033	peptidase M24	P:GO:0006508;<br>F:GO:0070006	P:proteolysis;<br>F:metalloaminopeptidase activity
9286	serine dehydratase	F:GO:0003941;<br>P:GO:0006094;<br>F:GO:0046872;<br>F:GO:0051539	F:L-serine ammonia-lyase activity;<br>P:gluconeogenesis;<br>F:metal ion binding;<br>F:4 iron, 4 sulfur cluster binding
9754	low-density lipoprotein receptor-related protein 6	P:GO:0001745;<br>C:GO:0005769;<br>C:GO:0005770;<br>C:GO:0005886;<br>P:GO:0007367;<br>P:GO:0007399;<br>P:GO:0007616;<br>P:GO:0008587;<br>F:GO:0015026;<br>C:GO:0016021;<br>F:GO:0017147;<br>P:GO:0035223;<br>F:GO:0042813;<br>P:GO:0048076;<br>P:GO:0060070;<br>P:GO:0090254	P:compound eye morphogenesis;<br>C:early endosome;<br>C:late endosome;<br>C:plasma membrane;<br>P:segment polarity determination;<br>P:nervous system development;<br>P:long-term memory;<br>P:imaginal disc-derived wing margin morphogenesis;<br>F:coreceptor activity;<br>C:integral component of membrane;<br>F:Wnt-protein binding;<br>P:leg disc pattern formation;<br>F:Wnt-activated receptor activity;<br>P:regulation of compound eye pigmentation;<br>P:canonical Wnt signaling pathway;<br>P:cell elongation involved in imaginal disc-derived wing morphogenesis
10105	GA14711, isoform A	C:GO:0005634;<br>P:GO:0006355;<br>F:GO:0043565	C:nucleus;<br>P:regulation of transcription, DNA-templated;<br>F:sequence-specific DNA binding
10178	Cadherin-related tumor suppressor	F:GO:0005509;<br>C:GO:0005886;<br>P:GO:0007156;<br>C:GO:0016021	F:calcium ion binding;<br>C:plasma membrane;<br>P:homophilic cell adhesion via plasma membrane adhesion molecules;<br>C:integral component of membrane

10285	segmentation polarity homeobox protein engrailed	P:GO:0000122;<br>F:GO:0000978;<br><br>F:GO:0001227;<br><br>C:GO:0005634;<br>P:GO:0007367;<br>P:GO:0007388;<br>P:GO:0007400;<br>P:GO:0007411;<br>P:GO:0007418;<br>P:GO:0007474;<br>P:GO:0007485;<br>P:GO:0007486;<br>P:GO:0007487;<br>P:GO:0008406;<br>P:GO:0021960;<br>P:GO:0035224;<br>P:GO:0035277;<br>P:GO:0035288;<br>P:GO:0035289;<br>P:GO:0035290;<br>P:GO:0043524;<br>P:GO:0045944;<br>P:GO:0048099;<br>P:GO:0048100	P:negative regulation of transcription by RNA polymerase II;<br>F:RNA polymerase II cis-regulatory region sequence-specific DNA binding;<br>F:DNA-binding transcription repressor activity, RNA polymerase II-specific;<br>C:nucleus;<br>P:segment polarity determination;<br>P:posterior compartment specification;<br>P:neuroblast fate determination;<br>P:axon guidance;<br>P:ventral midline development;<br>P:imaginal disc-derived wing vein specification;<br>P:imaginal disc-derived male genitalia development;<br>P:imaginal disc-derived female genitalia development;<br>P:analia development;<br>P:gonad development;<br>P:anterior commissure morphogenesis;<br>P:genital disc anterior/posterior pattern formation;<br>P:spiracle morphogenesis, open tracheal system;<br>P:anterior head segmentation;<br>P:posterior head segmentation;<br>P:trunk segmentation;<br>P:negative regulation of neuron apoptotic process;<br>P:positive regulation of transcription by RNA polymerase II;<br>P:anterior/posterior lineage restriction, imaginal disc;<br>P:wing disc anterior/posterior pattern formation
10330	purine nucleoside phosphorylase	F:GO:0004731;<br>P:GO:0009116	F:purine-nucleoside phosphorylase activity;<br>P:nucleoside metabolic process
12409	mediator of RNA polymerase II transcription subunit 14 isoform X2	F:GO:0003712;<br>P:GO:0006357;<br>C:GO:0016592	F:transcription coregulator activity;<br>P:regulation of transcription by RNA polymerase II;<br>C:mediator complex
12686	molybdenum ABC transporter permease	F:GO:0005524;<br>C:GO:0005886;<br>F:GO:0015098;<br>P:GO:0015689;<br>C:GO:0016021;<br>F:GO:0016887;<br>P:GO:0055085	F:ATP binding;<br>C:plasma membrane;<br>F:molybdate ion transmembrane transporter activity;<br>P:molybdate ion transport;<br>C:integral component of membrane;<br>F:ATPase activity;<br>P:transmembrane transport
13439	probable malonyl-CoA-acyl carrier protein transacylase, mitochondrial	P:GO:0008152;<br>F:GO:0016740	P:metabolic process;<br>F:transferase activity
13734	proclotting enzyme	F:GO:0004252;<br>P:GO:0006508;<br>P:GO:0009620	F:serine-type endopeptidase activity;<br>P:proteolysis;<br>P:response to fungus
15247	116 kDa U5 small nuclear ribonucleoprotein component	F:GO:0003924;<br>F:GO:0005525;<br>C:GO:0005737;<br>P:GO:0010628;<br>C:GO:0030532	F:GTPase activity;<br>F:GTP binding;<br>C:cytoplasm;<br>P:positive regulation of gene expression;<br>C:small nuclear ribonucleoprotein complex
15554	CG43901, isoform A	C:GO:0005634;<br>P:GO:0006355;<br>F:GO:0043565	C:nucleus;<br>P:regulation of transcription, DNA-templated;<br>F:sequence-specific DNA binding

16415	hypothetical protein FF38_06183	C:GO:0016020;<br>F:GO:0016798	C:membrane;<br>F:hydrolase activity, acting on glycosyl bonds
16695	hypothetical protein FF38_06816	F:GO:0003700;<br>F:GO:0005509;<br>C:GO:0005634;<br>P:GO:0006355;<br>F:GO:0008270;<br>C:GO:0016021	F:DNA-binding transcription factor activity;<br>F:calcium ion binding;<br>C:nucleus;<br>P:regulation of transcription, DNA-templated;<br>F:zinc ion binding;<br>C:integral component of membrane
16903	Ig-like and fibronectin type-III domain-containing protein C25G4.10	F:GO:0005515;<br>C:GO:0016021	F:protein binding;<br>C:integral component of membrane
17088	roundabout homolog 2	P:GO:0003151;<br>C:GO:0005886;<br>P:GO:0007432;<br>P:GO:0008045;<br>F:GO:0008201;<br>P:GO:0008406;<br>P:GO:0009649;<br>C:GO:0016021;<br>P:GO:0016199;<br>P:GO:0022409;<br>C:GO:0030425;<br>C:GO:0031982;<br>P:GO:0035050;<br>P:GO:0035385;<br>C:GO:0043025;<br>C:GO:0044295;<br>P:GO:0046716;<br>P:GO:0050773;<br>P:GO:0070983;<br>C:GO:0071666;<br>P:GO:0072499;<br>P:GO:2000274	P:outflow tract morphogenesis;<br>C:plasma membrane;<br>P:salivary gland boundary specification;<br>P:motor neuron axon guidance;<br>F:heparin binding;<br>P:gonad development;<br>P:entrainment of circadian clock;<br>C:integral component of membrane;<br>P:axon midline choice point recognition;<br>P:positive regulation of cell-cell adhesion;<br>C:dendrite;<br>C:vesicle;<br>P:embryonic heart tube development;<br>P:Roundabout signaling pathway;<br>C:neuronal cell body;<br>C:axonal growth cone;<br>P:muscle cell cellular homeostasis;<br>P:regulation of dendrite development;<br>P:dendrite guidance;<br>C:Slit-Robo signaling complex;<br>P:photoreceptor cell axon guidance;<br>P:regulation of epithelial cell migration, open tracheal system
17246	ADP-heptose--LPS heptosyltransferase II	P:GO:0008152;<br>F:GO:0016757	P:metabolic process;<br>F:transferase activity, transferring glycosyl groups
17326	protein hunchback isoform X1	F:GO:0003676	F:nucleic acid binding
17402	glutaconyl-CoA decarboxylase subunit beta	P:GO:0006814;<br>C:GO:0016021;<br>F:GO:0018801	P:sodium ion transport;<br>C:integral component of membrane;<br>F:glutaconyl-CoA decarboxylase activity
17565	aminopeptidase	F:GO:0004177;<br>P:GO:0006508;<br>F:GO:0008237;<br>F:GO:0008270	F:aminopeptidase activity;<br>P:proteolysis;<br>F:metallopeptidase activity;<br>F:zinc ion binding
18085	CG32206, isoform E	C:GO:0016021	C:integral component of membrane
18135	slit homolog 2 protein	C:GO:0016021	C:integral component of membrane
18326	filamin-C isoform X1	F:GO:0003779;<br>P:GO:0009612;<br>P:GO:0048104;<br>P:GO:0072499	F:actin binding;<br>P:response to mechanical stimulus;<br>P:establishment of body hair or bristle planar orientation;<br>P:photoreceptor cell axon guidance
18639	DnaJ domain-containing protein, putative	F:GO:0005102;<br>C:GO:0016021	F:signaling receptor binding;<br>C:integral component of membrane

18798	isoamylase	F:GO:0003824;<br>P:GO:0005975	F:catalytic activity;<br>P:carbohydrate metabolic process
19868	PREDICTED: agrin	C:GO:0016021	C:integral component of membrane
20396	LexA family transcriptional regulator	F:GO:0003677;<br>F:GO:0004252;<br>P:GO:0006260;<br>P:GO:0006281;<br>P:GO:0006508;<br>P:GO:0009432;<br>P:GO:0045892	F:DNA binding;<br>F:serine-type endopeptidase activity;<br>P:DNA replication;<br>P:DNA repair;<br>P:proteolysis;<br>P:SOS response;<br>P:negative regulation of transcription, DNA-templated
20670	adenylate cyclase type 2	F:GO:0000166;<br>F:GO:0004016;<br>C:GO:0005622;<br>C:GO:0005886;<br>P:GO:0006171;<br>C:GO:0016021;<br>P:GO:0035556	F:nucleotide binding;<br>F:adenylate cyclase activity;<br>C:intracellular;<br>C:plasma membrane;<br>P:cAMP biosynthetic process;<br>C:integral component of membrane;<br>P:intracellular signal transduction
20738	lipoyl synthase	C:GO:0005737;<br>P:GO:0009107;<br>P:GO:0009249;<br>F:GO:0016992;<br>F:GO:0046872;<br>F:GO:0051539	C:cytoplasm;<br>P:lipoate biosynthetic process;<br>P:protein lipoylation;<br>F:lipoate synthase activity;<br>F:metal ion binding;<br>F:4 iron, 4 sulfur cluster binding
20973	hypothetical protein FF38_05889	F:GO:0003676;<br>C:GO:0016021;<br>F:GO:0016787;<br>F:GO:0046872	F:nucleic acid binding;<br>C:integral component of membrane;<br>F:hydrolase activity;<br>F:metal ion binding
21327	zinc transporter 9	F:GO:0008324;<br>C:GO:0016021;<br>P:GO:0098655	F:cation transmembrane transporter activity;<br>C:integral component of membrane;<br>P:cation transmembrane transport
22150	SWI/SNF complex subunit SMARCC2 isoform X2	F:GO:0003677;<br>F:GO:0005515;<br>P:GO:0006357;<br>P:GO:0007406;<br>P:GO:0007474;<br>P:GO:0007480;<br>P:GO:0008586;<br>P:GO:0008587;<br>C:GO:0016514;<br>C:GO:0016586;<br>C:GO:0035060;<br>P:GO:0043044;<br>P:GO:0045088;<br>P:GO:0045893;<br>C:GO:0071564;<br>C:GO:0071565;<br>P:GO:2000134	F:DNA binding;<br>F:protein binding;<br>P:regulation of transcription by RNA polymerase II;<br>P:negative regulation of neuroblast proliferation;<br>P:imaginal disc-derived wing vein specification;<br>P:imaginal disc-derived leg morphogenesis;<br>P:imaginal disc-derived wing vein morphogenesis;<br>P:imaginal disc-derived wing margin morphogenesis;<br>C:SWI/SNF complex;<br>C:RSC-type complex;<br>C:brahma complex;<br>P:ATP-dependent chromatin remodeling;<br>P:regulation of innate immune response;<br>P:positive regulation of transcription, DNA-templated;<br>C:npBAF complex;<br>C:nBAF complex;<br>P:negative regulation of G1/S transition of mitotic cell cycle
214095	Alpha-L-fucosidase	F:GO:0004560;<br>P:GO:0006004	F:alpha-L-fucosidase activity;<br>P:fucose metabolic process
226347	glutathione S-transferase	F:GO:0004364	F:glutathione transferase activity

Supplementary Table S2. Pairwise linkage disequilibrium between microsatellite loci. Statistically significant differences are indicated by asterisks: * $p < 0.05$, ** $p < 0.006$.

		P-value	
**Locus 1**	**Locus 2**	**2015 Sample**	**2016 Sample**
CH01	CH05	0.586370	0.586170
CH01	CH09	0.160350	0.034380*
CH05	CH09	0.454380	0.838580
CH01	CH12	1.000000	0.000000**
CH05	CH12	0.886270	0.124870
CH09	CH12	0.770180	0.000360**
CH01	CH14	0.220510	0.000000**
CH05	CH14	0.638530	0.119450
CH09	CH14	0.251030	0.536360
CH12	CH14	0.987650	0.002780**
CH01	CH15	0.374290	0.010970*
CH05	CH15	0.899110	0.542880
CH09	CH15	0.963080	0.259410
CH12	CH15	1.000000	0.246800
CH14	CH15	0.810280	0.327000
CH01	CH24	1.000000	0.029460*
CH05	CH24	0.659180	0.383390
CH09	CH24	0.843900	0.053430
CH12	CH24	0.543510	0.089310
CH14	CH24	1.000000	0.220070
CH15	CH24	0.368190	0.421300
CH01	CH26	0.175640	0.002990**
CH05	CH26	0.959870	0.007710*
CH09	CH26	0.864760	0.000000**
CH12	CH26	1.000000	0.000000**
CH14	CH26	0.623260	0.187100
CH15	CH26	0.401790	0.578220
CH24	CH26	0.742220	0.000000**
